# Genetic Analysis of Litter Size Across Parities in Prolific and Conventional Populations of Tunisian Barbarine Sheep Using a Random Regression Model

**DOI:** 10.3390/ani15050638

**Published:** 2025-02-22

**Authors:** Chiraz Ziadi, Juan Manuel Serradilla, Sonia Bedhiaf-Romdhani, Antonio Molina

**Affiliations:** 1 Departamento de Genética, Universidad de Córdoba, Campus de Rabanales, Ctra, Madrid-Cádiz, km 396, 14071 Córdoba, Spain; ge1moala@uco.es; 2 Departamento de Producción Animal, Universidad de Córdoba, Campus de Rabanales, Ctra, Madrid-Cádiz, km 396, 14071 Córdoba, Spain; jmserradilla@gmail.com; 3 Laboratoire des Productions Animales et Fourragères, Institut National de la Recherche Agronomique de Tunisie, Rue Hédi Karray, Ariana 2049, Tunisia; bedhiaf.sonia@gmail.com

**Keywords:** random regression model, Legendre polynomials, litter size, parity, sheep

## Abstract

Litter size records from two lines of Tunisian Barbarine sheep were analysed across parities using an RRM. A total of 2751 and 2562 litter records from the first to the sixth parity from the prolific and the conventional lines, respectively, were included in the analysis. The total number of animals in the pedigree was 1277 for the prolific line and 1102 for the conventional line. The estimation of genetic parameters was based on Bayesian inference under categorical distribution. Fixed effects included the year and month of lambing and a fixed quadratic regression coefficient for the lambing number with Legendre polynomials. The random additive and permanent environmental effects were modelled by second-order Legendre polynomials. Heritability ranged from 0.04 to 0.18 for the prolific line and from 0.17 to 0.39 for the conventional line. Genetic correlations within trait through parities showed a wide range of values, from 0.25 to 0.96 for the prolific line and from zero to 0.93 for the conventional line. Due to the changes in the variances and the genetic correlations different from unity across parities, the use of an RRM is recommended to analyse litter size in the Barbarine sheep.

## Simple Summary

The Barbarine is an autochthonous sheep breed originating from Tunisia and raised in low-input production systems. This study was conducted to estimate the genetic parameters of litter size in two lines of this Barbarine breed using a random regression model (RRM). An RRM is recommended to better model the change in variance structure over time and to obtain a more accurate genetic evaluation of traits that are repeatedly measured over time. Estimates of variance components were not constant across parities in either line. In addition, within-trait genetic correlations across parities were different from unity. Thus, litter size should be treated as a distinct trait throughout the life of a female in the Barbarine breed. This study provided valuable insights for optimising breeding programmes for these two lines to accelerate genetic progress, which is crucial in low-input production systems.

## 1. Introduction

The Barbarine fat-tailed breed is the main meat sheep raised in Tunisia in low-input production systems [1]. This breed is characterised by its adaptation to the harsh environmental conditions prevailing in the country [2]. A prolificacy-based selection programme has been implemented since 1979 by the Tunisian National Institute for Agronomic Research (INRAT) in the research centre of Oueslatia, Kairouan, creating a line of prolific ewes of the fat-tailed Barbarine sheep. The non-prolific animals were selected for growth, resulting in the creation of the conventional line [1]. Both lines are raised at the same research centre of Oueslatia in the same low-input production system.

The Barbarine sheep is a low-prolific breed [3] due to the large heterogeneity of environmental conditions [4] and most females give birth to single litters. In this context, increasing the litter size per breeding female can effectively improve the overall economic profitability of this breed, where the majority of income is derived from the sale of lambs.

In animal breeding, many traits of interest are measured repeatedly over the lifetime of an individual, resulting in longitudinal data. The models commonly used to analyse these types of data are repeatability (RM), multiple trait models (MTMs) and random regression models (RRMs) [5]. The repeatability model is often used because of its simplicity and reduced number of parameters to estimate compared to a multiple-trait or random regression model. However, this model assumes that genetic correlations between measurements of the trait at different times are equal to unity and that the variance is constant over the genetic trajectory [5,6]. In the MTM, measurements at different time points are considered as multiple response variables. The main advantage of using an MTM over an RM is the improved accuracy of the evaluation for each trait due to better connections in the data between the genetic and residual covariance [5,7]. However, a high correlation between consecutive measurements and computational requirements may limit the application of the multiple-trait model [8]. The RRM has been used to predict the change in a trait continuously over time with fewer parameters than the multiple-trait model. The advantages of using an RRM over RMs and MTMs include its flexibility in modelling individual-specific trajectories and estimating covariance structure and breeding values at any point along the trait trajectory and the reduction in the number of parameters to be estimated [6,9]. The RRM also improves the accuracy of parameter estimation [10,11] and provides a better handling of missing data and the incorporation of within-trait genetic correlations over time [12,13].

Litter size is measured multiple times over the lifetime of an animal, and although it differs from longitudinal traits because the parity number is a discrete variable, the application of an RRM to the analysis of this trait is still feasible [14]. Genetic analyses of litter size have been performed using repeatability [15,16,17], multiple-trait [18,19,20] or RRMs [21,22,23]. In sheep, genetic parameters for litter size are only available with repeatability or multiple-trait models [24,25].

Therefore, the main objective of this study was to estimate, for the first time, genetic parameters and their changes across parities for litter size in prolific and conventional populations of Barbarine sheep using a threshold random regression model.

## 2. Materials and Methods

### 2.1. Data and Pedigree

This study was based on two lines of the Tunisian Barbarine meat sheep breed. The first population is a prolific line selected for increased litter size, while the second is a conventional line selected for weaning weight. Both lines are raised under the same management and environmental conditions in a low-input production system at the Ouesslatia research station, located in a semi-arid climate in the centre–east of Tunisia. For both breeds, mating occurs in the spring using the ram effect, and lambing takes place between September and November. Reproduction is managed through seven families where one ram and its replacement are assigned for each family. For mating, females born in a given family are systematically moved to another family to limit inbreeding. The weaning of lambs occurs at approximately 3 months of age.

The rearing system is mainly based on natural pasture with the supplementation of hay and concentrates during mating, late gestation and the beginning of the suckling period. Details on the genetic origin and breeding scheme of these two lines are available in the synthesis article of Bedhiaf et al. [26]. The reproductive and genealogical information of the two populations was provided by the Tunisian National Institute for Agronomic Research (INRAT) research station of Ouesslatia, Tunisia. The reproductive data records consisted of ewe identification, ewe birth year, lambing number, lambing date, and the total number of lambs born. The pedigree information included animal identification, date of birth, sex, and the animal’s sire and dam. A total of 2751 records from 992 ewes of the prolific line collected over 29 years (an average of 2.77 lambings per female) and 2562 records from 857 ewes of the conventional line collected over 19 years (an average of 2.98 lambings per female) were used in this analysis. Litter size (LS) was estimated as the total number of lambs born and was recorded on the day of lambing from the first to the sixth parity in both lines. The pedigree was traced back for as many generations as were available in the line’s herd books. At least 3 complete generations and 4.6 equivalent generations were included in the pedigree, giving a total of 1277 animals for the prolific line and 1102 animals for the conventional line.

### 2.2. Statistical Analysis

In a preliminary analysis using the ‘GLM2’ R package [27], the significance of the non-genetic effects for the year of lambing and month of lambing of the ewe and their inclusion in the model were determined. All of them had a significant effect on litter size at the 0.05 significance level.

Analyses were performed separately for the prolific and conventional lines by fitting a univariate threshold random regression model for LS. To deal with the non-normal distribution of LS, this trait was treated as a threshold trait, with a multinomial distribution (three categories: one, two, and more than two) for the prolific line and a binomial distribution (two categories: one and more than one) for the conventional line.

LS is measured at various parities throughout the female’s life, making it possible to apply an RRM with Legendre orthogonal polynomials that accounts for the variation in genetic parameters over time. Orthogonal Legendre polynomials are non-parametric functions used to model the variances and covariances of longitudinal traits, often standardised in the interval from −1 to +1.

The analysis was performed using the following model for both lines:

$$y_{ijlt}={YL}_{i}+{ML}_{j}+\sum_{k=0}^{2}\beta_{k}Z_{tk}+\sum_{k=0}^{2}\alpha_{ltk}Z_{tk}+\sum_{k=0}^{2}\mu_{ltk}Z_{tk}+e_{ijlt}$$

where $y_{ijlt}$ is a vector with n observations of LS, ${YL}_{i}$ is the fixed effect of the year of lambing (29 and 19 levels for the prolific and conventional lines, respectively), ${ML}_{j}$ is the fixed effect of the month of lambing (3 levels for both lines, from September to November), and $\beta_{k}$ are k coefficients of the fixed regression of the LS trajectory in females over parities with orthogonal Legendre polynomials of the second order. Six parity ranges were considered as the unit of time t (t = 1–5 and t ≥ 6) and their effects were considered as a continuous variable expressed in a standardised form between −1 and +1 according to the expression of Kirkpatrick et al. [9]. $Z_{tk}$ is the k^th^ Legendre polynomial corresponding to parity t, assumed to be the same for fixed and random coefficients. The terms $\alpha_{ltk}$ and $\mu_{ltk}$ are the k^th^ random regression coefficients associated with the additive genetic function and the permanent environmental function of female l in parity t, respectively, and $e_{ijlt}$ is the residual effect modelled with heterogeneous variance according to parity with three measurements for the prolific line (parity 1, 2 to 4, and 5 and 6) and the conventional line (parity 1, 2 and 6 and 3 to 5).

In matrix notation, the RRM is as follows:

y=Xb+Za+Wp+e

where y is a vector of observations for LS, b contains fixed effects previously defined and fixed regression coefficients, a contains genetic additive regression coefficients of each animal, p contains permanent environmental regression coefficients of each female with LS data, and e is a vector of residual effects. X, Z and W are incidence and covariate matrices relating observations to b, a and p, respectively.

In this RRM, the expected (co)variance components are assumed to be as follows:

$$var\left[\begin{array}{c} a\\ p\\ e \end{array}\right]=\left[\begin{array}{ccc} A⊗G & 0 & 0\\ 0 & I⊗P & 0\\ 0 & 0 & R \end{array}\right]$$

where G is the (co)variance matrix of the additive genetic random regression coefficients, assumed to be the same for all animals, P is the (co)variance matrix of the permanent environmental random regression coefficients, R is a diagonal matrix of residual variances, A is the relationship matrix among all animals in the pedigree, I is the identity matrix, and ⊗ is the Kronecker product. In addition, a univariate threshold repeatability animal model as described by Ziadi et al. [1] was applied for LS in the prolific and conventional lines.

The estimation of all (co)variance components was performed by Bayesian inference using the THRGIBBS3F90 software [28]. Flat priors were assumed for systematic effects of the year of lambing and month of lambing, as well as for additive genetic, permanent environmental, and residual effects. Chains of 500,000 samples were used, with a burn-in period of 100,000. One sample was saved every one hundred samples to avoid high correlations between consecutive samples. The remaining 4000 samples were used to compute the posterior means of the model parameters through a post-Gibbs analysis based on the POSTGIBBSf90 software [29].

The (co)variance components, heritabilities ( $h^{2}$ ), and genetic correlations ( $r_{g}$ ) over the range of the parity of females were estimated using the expression proposed by Jamrozik and Schaeffer [13].

## 3. Results and Discussion

To date, no studies have estimated the genetic parameters for litter size using a random regression model in sheep. Previous studies on the genetic analysis of litter size have usually used repeatability and multiple-trait approaches. Therefore, in this study, we propose, for the first time in sheep, an RRM that takes into account the within-trait correlations over time to analyse litter size. The results of this study demonstrated that the RRM effectively captured the genetic variability and within-trait correlations over time for litter size, thereby achieving the objective of providing a more comprehensive genetic analysis in the Barbarine breed.

### 3.1. Phenotypic Performances

A summary of the descriptive statistics for litter size in both lines is presented in Table 1.

The overall phenotypic means and standard deviations of LS were 1.56 ± 0.62 and 1.07 ± 0.26 lambs/ewe for the prolific line and conventional line, respectively. These means varied from one parity to another. The maximum values of LS were observed in the fourth parity in the prolific line and in the first parity in the conventional line. The phenotypic trajectory of LS reflected the higher reproductive performance of the prolific line and its greater persistence, with an increase until the fourth parity, followed by a decrease, with values remaining high compared to the beginning of the reproductive life of the female. On the contrary, this decline started from the first parity and continued until the last parity in the conventional line. This outcome could be attributed to the strategy that has been adopted for the prolific line since the 1970s, which is based on the selection of ewe lambs born from large litters and the more rustic (less improved) nature of the conventional line. In addition, the genotyping of a sample of individuals from this prolific line revealed the presence of a mutation called FecX+/FecXBar in the bone morphogenetic protein 15 (BMP15/FecX) gene [30,31]. In this same line, the frequency of the FecXBar carrier females was 0.42 (39% of heterozygous carriers and 3% of homozygous carriers) [31].

The BMP15 gene was first described by Davis et al. [32] in Romney sheep and named the Inverdale gene. It carries a naturally occurring X-linked mutation that causes an increased ovulation rate and twin and triplet births in heterozygotes but primary ovarian failure and sterility in homozygotes [33]. To date, in the BMP15 gene, approximately 20 different mutations have been identified in other sheep breeds around the world with different effects on ovulation rate and litter size [34]. For example, Altarriba et al. [35] reported an average LS of 1.35 ± 0.48 in the Rasa Aragonesa, and Martin et al. [36] and Chantepie et al. [37] observed higher values in the Mouton Vendéen breed and Belclare ewes (2.1 and 1.72, respectively).

The differences observed between the breeds could be due to the different mutations identified, the frequency of the prolific allele and its association with high LS, its inclusion in the prolificity management of the population, and the genetic structure and breeding conditions of the breeds.

The mean LS in the conventional line was similar to the values observed in several conventional flocks of the Barbarine breed (1.08 ± 0.23) [31]. Nevertheless, the Noire de Thibar, raised in a semi-intensive production system, and the Queue-fine de l’ouest flock, located in a semi-arid environment, showed higher LS, 1.32 ± 0.47 and 1.37 ± 0.56, respectively [38,39].

The coefficient of variation was estimated at 40.03% for the prolific line and 24.06% for the conventional line. This indicated a high phenotypic variability in both populations, with greater variability observed in the prolific line due to the occurrence of multiple births and the fact that hyperprolific females are more susceptible to adverse environmental conditions than conventional females. In their meta-analysis in different breeds (wool, dual-purpose, and meat), Fogarty [40] and Safari et al. [41] reported a CV for litter size of 34.1 ± 0.6 and 36%, respectively.

### 3.2. Genetic Parameters

#### 3.2.1. Variance Components and Heritability

The RRM is used to estimate genetic parameters because it allows us to study the variation in the trait to be studied as a function of time or, in the case of this study, parities. Therefore, finding the optimal order of fit in the RRM is crucial. In the current study, different orders of polynomials were tested, from the first to the fifth one, and the optimum was the second order, selected on the basis of the Akaike information criterion. For this reason, only the results of the second-order Legendre polynomial RRM are presented in this study. In the same way, several studies have shown that the second order of fit is adequate to model the variation for the genetic and permanent environmental variances for litter size over time for other species [22,23,42,43].

The results of the variance components and heritabilities for LS using a repeatability model and over the six parities of females using a random regression model are showed in Table 2 and Table 3 for the prolific line and conventional line, respectively.

All these parameters were presented on a liability scale. In both lines, the variance components for the additive genetic and permanent environmental effects were not constant across the range of parities in the ewes, indicating that, from a genetic point of view, LS is not controlled by the same genes at different parities and should not be considered as the same trait throughout the life of the female.

In the prolific line, the additive genetic variances and heritabilities increased from the first to the last parity and the $h^{2}$ varied between 0.04 in the first parity and 0.18 in the last parity (Table 2). The permanent environmental variance was higher in the first two and last parities and was significantly greater than the additive genetic variances in the first two parities, indicating that the permanent environmental effects had a greater influence on the variation in litter size at the beginning and end of the reproductive life of the female.

In the conventional line, the additive genetic variances and heritabilities decreased from the first to the second parity and then increased continuously until the last parity, where the highest values were observed. Estimates of $h^{2}$ ranged from 0.17 in the second parity to 0.39 in the last parity (Table 3). The permanent environmental variances were high in the first and last parity and were greater than the additive genetic variances in all parities.

In the repeatability model, the $h^{2}$ was similar in both lines (0.14 for the prolific line and 0.15 for the conventional line). The additive genetic variance estimated by the RM or RRM across parities was lower in the prolific line than in the conventional line. Similarly, the deviation of the additive genetic variance for each parity, obtained with the RRM, relative to the additive genetic variance estimated in the RM, was notably smoother in the prolific line. This could be explained by the fact that in the parity trajectory of females from the prolific line, the expression of the major *BMP15* gene, with its strong and dominant effect, may account for a significant portion of the genetic variance, leaving less opportunity for other additive genes to contribute to the trait, thereby reducing variability across parities. In contrast, in the case of the conventional line, the genetic variance is purely polygenic, driven by genes with small effects whose expression can vary throughout the life of the animals.

Litter size in sheep has usually been analysed using linear models. Canario et al. [44] reported that the use of linear models rather than threshold models to analyse categorical traits could lead to some bias. In fact, the use of the threshold model resulted in an increase in the heritability of LS [45,46].

It has been reported in the literature that the heritability of litter size is generally low, as expected for reproductive traits and elsewhere in sheep [40,41]. Estimates of $h^{2}$ in the prolific line were similar to those reported in previous studies in the same line using a linear repeatability model [26,47] and, more recently, using both linear and threshold repeatability models [1]. In general, our $h^{2}\;$ was within the range of estimates reported in many studies with linear and threshold models in prolific breeds, where major genes controlling prolificacy have been identified [35,36,48].

The fraction of phenotypic variance explained by permanent environmental variance ( $c^{2}$ ) obtained with the RRM varied from 0.08 to 0.41 (Table 2) in the prolific line (a mean of 0.18) and from 0.29 to 0.49 (Table 3) in the conventional line (a mean of 0.34). Estimates of $c^{2}$ were not constant across the parity trajectory in both lines, suggesting that non-additive genetic or permanent environmental effects may not influence LS uniformly throughout the reproductive life of the sheep.

#### 3.2.2. Phenotypic and Genetic Correlations

Estimates of within-trait phenotypic ( $r_{p}$ ) and genetic ( $r_{g}$ ) correlations for LS across the six parities in the prolific and conventional lines are provided in Table 4 and Table 5, respectively.

Estimates of phenotypic correlations between LSs across different parities were low, ranging from almost zero to 0.23 in the prolific line and from almost zero to 0.30 in the conventional line.

Estimates of genetic correlations between LSs at different parities were all positive, ranging from medium (0.25 between the second and sixth parity groups, Table 4) to high (0.96 between the third and fourth parity groups, Table 4) for the prolific line. In the conventional line, $r_{g}$ ranged from zero for the first and fourth parity groups to 0.93 for the third and fourth parity groups of the diagonal (Table 5). Incomplete within-trait genetic correlations at different parities indicate that LS records along the female lifetime should be treated as different traits [22,49,50].

In all cases, these estimates were high between adjacent parities (near the diagonal) and close to unity, except for $r_{g}$ between the first and second parities in both lines. The estimates decreased as the interval between parities increased, with the exception of $r_{g}$ between the first and sixth parities in the prolific line and $r_{g}$ between the first, fifth, and sixth parities in the conventional line. The decrease in within-trait genetic correlation with increasing intervals between measurements (parities) is a result frequently reported for the RRM in the literature [19,23,51,52,53]. This could be attributed to the limited amount of data available in later parities and the imprecise recording of parity order [22].

The conventional and prolific lines originate from the same base population and are raised under the same low-input production system. However, since the discovery of the presence of hyperprolific ewes in this population, different breeding strategies have been adopted for each of them. A prolificacy-based selection programme was implemented for the prolific line, while the conventional line was selected to enhance weaning weight. Therefore, we consider that it would have been interesting to have been able to obtain common ancestors for both populations in order to refine the comparison of both lines. The fact that this separation took place in the 1970s of the last century, and that many generations have passed, has prevented us from having these common ancestors.

Recently, a growing body of interest was poured into the improvement of reproductive traits in meat sheep flocks by the introgression of the prolificacy gene into conventional flocks, and the preliminary results have already been published [54]. This approach will facilitate the dissemination of fecundity genes and will contribute to livelihood improvement in communities raising Barbarine sheep.

Finally, based on the estimates of genetic parameters and genetic correlations provided by this work, it is justified to consider litter traits at different parities of ewes as distinct traits. The heterogeneity of the variance components for all litter traits at different parities and the correlations between parities, which are substantially lower than unity, further support the use of the RRM in the genetic evaluation of selection candidates in the Barbarine breed.

## 4. Conclusions

This study aimed to determine whether litter size across different parities in the Barbarine breed could be considered a single trait for genetic evaluation, and the results obtained sufficiently address this objective. In conclusion, due to the differences in the estimates of variance components along the scale of parity values observed in both lines, and the genetic correlations different from unity, litter size in the different parities throughout the life of a ewe cannot be treated as the same trait in genetic assessments. Therefore, the use of an RRM to analyse litter size is recommended for the breeding programme of the Barbarine breed. Further studies could continue by estimating the genetic progress that has occurred in growth and prolificacy in both populations.

## Figures and Tables

**Table 1:** Descriptive statistics for litter size (LS) across parities in the prolific and the conventional Barbarine lines.

	Prolific					Conventional				
Parity	N	Mean ± SD	Min	Max	CV (%)	N	Mean ± SD	Min	Max	CV (%)
1	813	1.43 ± 0.57	1.0	4.0	39.86	857	1.08 ± 0.28	1.0	3.0	25.93
2	681	1.49 ± 0.60	1.0	4.0	40.26	566	1.07 ± 0.25	1.0	2.0	23.36
3	434	1.63 ± 0.62	1.0	4.0	38.03	446	1.06 ± 0.24	1.0	3.0	22.64
4	316	1.72 ± 0.61	1.0	4.0	35.46	313	1.07 ± 0.25	1.0	2.0	23.36
5	240	1.67 ± 0.69	1.0	4.0	41.31	205	1.04 ± 0.21	1.0	2.0	20.19
6	267	1.65 ± 0.66	1.0	4.0	40.00	175	1.03 ± 0.18	1.0	2.0	17.48
All parities	2751	1.56 ± 0.62	1.0	4.0	40.03	2562	1.07 ± 0.26	1.0	3.0	24.06

N: number of records; SD: standard deviation; Min: minimum; Max: maximum; CV: coefficient of variation.

**Table 2:** Estimates of genetic parameters for litter size (LS) across parities in the prolific Barbarine line using a threshold repeatability and random regression models.

Model	Parity	$\sigma_{a}^{2}$	$\sigma_{p}^{2}$	$\sigma_{e}^{2}$	$h^{2}$	HPD_95%_	P	k	$c^{2}$
Repeatability	-	0.060	0.043	0.306	0.14	0.05, 0.24	0.81	0.06	0.10
Random regression	1	0.060	0.582	0.769	0.04	0.0009, 0.11	0.08	0.005	0.41
	2	0.065	0.219	0.735	0.06	0.003, 0.13	0.17	0.012	0.21
	3	0.104	0.110	0.735	0.11	0.02, 0.21	0.53	0.033	0.12
	4	0.119	0.076	0.735	0.13	0.029, 0.22	0.68	0.046	0.08
	5	0.130	0.085	0.794	0.13	0.02, 0.23	0.67	0.036	0.08
	6	0.231	0.251	0.794	0.18	0.006, 0.35	0.76	0.033	0.20

$\sigma_{a}^{2}$ : additive genetic variance; $\sigma_{p}^{2}$ : permanent environmental variance; $\sigma_{e}^{2}$ : residual variance; $h^{2}$ : heritability; HPD_95%_: high posterior density interval at 95%; P: probability of the heritability being greater than 0.10; k: limit for the interval [k, +1) of the heritability with a probability of 95%; $c^{2}$ : fraction of phenotypic variance explained by the permanent environmental effect.

**Table 3:** Estimates of genetic parameters for litter size (LS) across parities in the conventional Barbarine line using a threshold repeatability model and random regression models.

Model	Parity	$\sigma_{a}^{2}$	$\sigma_{p}^{2}$	$\sigma_{e}^{2}$	$h^{2}$	HPD_95%_	P	k	$c^{2}$
Repeatability	-	0.223	0.273	1.003	0.15	0.0001, 0.3035	0.66	0.012	0.18
Random regression	1	0.822	1.768	1.003	0.23	0.005, 0.55	0.75	0.027	0.49
	2	0.338	0.577	1.066	0.17	0.0003, 0.39	0.68	0.011	0.29
	3	0.387	0.635	1.003	0.19	0.015, 0.39	0.76	0.044	0.31
	4	0.422	0.738	1.003	0.20	0.006, 0.42	0.72	0.029	0.34
	5	0.526	0.656	1.003	0.24	0.003, 0.51	0.73	0.015	0.30
	6	1.406	1.130	1.066	0.39	0.05, 0.71	0.94	0.092	0.31

$\sigma_{a}^{2}$ : additive genetic variance; $\sigma_{p}^{2}$ : permanent environmental variance; $\sigma_{e}^{2}$ : residual variance; $h^{2}$ : heritability; HPD_95%_: high posterior density interval at 95%; P: probability of the heritability being greater than 0.10; k: limit for the interval [k, +1) of the heritability with a probability of 95%; $c^{2}$ : fraction of phenotypic variance explained by the permanent environmental effect.

**Table 4:** Estimates of genetic (above diagonal) and phenotypic correlations (below diagonal) between litter size (LS) across parities in the prolific Barbarine line using a threshold random regression model.

Parity	Parity					
	1	2	3	4	5	6
1	-	0.62	0.33	0.26	0.31	0.35
2	0.17	-	0.94	0.86	0.65	0.25
3	0.13	0.22	-	0.96	0.75	0.28
4	0.09	0.19	0.23	-	0.89	0.49
5	0.04	0.10	0.14	0.17	-	0.83
6	0.00	-0.006	0.02	0.08	0.15	-

**Table 5:** Estimates of genetic (above diagonal) and phenotypic correlations (below diagonal) between litter size (LS) across parities in the conventional Barbarine line using a threshold random regression model.

Parity	Parity					
	1	2	3	4	5	6
1	-	0.70	0.17	0.00	0.13	0.33
2	0.30	-	0.81	0.62	0.38	0.10
3	0.19	0.27	-	0.93	0.57	0.04
4	0.15	0.23	0.19	-	0.80	0.31
5	0.09	0.12	0.11	0.13	-	0.81
6	0.03	-0.05	-0.03	0.04	0.19	-

## Data Availability

The data are available from the corresponding author on reasonable request.
